# Social Support and Common Dyadic Coping in Couples' Dyadic Management of Type II Diabetes: Protocol for an Ambulatory Assessment Application

**DOI:** 10.2196/13685

**Published:** 2019-10-04

**Authors:** Janina Lüscher, Tobias Kowatsch, George Boateng, Prabhakaran Santhanam, Guy Bodenmann, Urte Scholz

**Affiliations:** 1 Applied Social and Health Psychology Department of Psychology University of Zurich Zurich Switzerland; 2 Institute of Technology Management University of St. Gallen St. Gallen Switzerland; 3 Department of Management, Technology, and Economics ETH Zurich Zurich Switzerland; 4 Clinical Psychology for Children/Adolescents and Couples/Families Department of Psychology University of Zurich Zurich Switzerland; 5 University Research Priority Program “Dynamics of Healthy Aging” University of Zurich Zurich Switzerland

**Keywords:** social support, common dyadic coping, type II diabetes, dyadic illness management, couples, mobile sensing, multimodal sensor data, ambulatory assessment application, MobileCoach, study protocol

## Abstract

**Background:**

Type II diabetes mellitus (T2DM) is a common chronic disease. To manage blood glucose levels, patients need to follow medical recommendations for healthy eating, physical activity, and medication adherence in their everyday life. Illness management is mainly shared with partners and involves social support and common dyadic coping (CDC). Social support and CDC have been identified as having implications for people’s health behavior and well-being. Visible support, however, may also be negatively related to people’s well-being. Thus, the concept of invisible support was introduced. It is unknown which of these concepts (ie, visible support, invisible support, and CDC) displays the most beneficial associations with health behavior and well-being when considered together in the context of illness management in couple’s everyday life. Therefore, a novel ambulatory assessment application for the open-source behavioral intervention platform MobileCoach (AAMC) was developed. It uses objective sensor data in combination with self-reports in couple’s everyday life.

**Objective:**

The aim of this paper is to describe the design of the Dyadic Management of Diabetes (DyMand) study, funded by the Swiss National Science Foundation (CR12I1_166348/1). The study was approved by the cantonal ethics committee of the Canton of Zurich, Switzerland (Req-2017_00430).

**Methods:**

This study follows an intensive longitudinal design with 2 phases of data collection. The first phase is a naturalistic observation phase of couples’ conversations in combination with experience sampling in their daily lives, with plans to follow 180 T2DM patients and their partners using sensor data from smartwatches, mobile phones, and accelerometers for 7 consecutive days. The second phase is an observational study in the laboratory, where couples discuss topics related to their diabetes management. The second phase complements the first phase by focusing on the assessment of a full discussion about diabetes-related concerns. Participants are heterosexual couples with 1 partner having a diagnosis of T2DM.

**Results:**

The AAMC was designed and built until the end of 2018 and internally tested in March 2019. In May 2019, the enrollment of the pilot phase began. The data collection of the DyMand study will begin in September 2019, and analysis and presentation of results will be available in 2021.

**Conclusions:**

For further research and practice, it is crucial to identify the impact of social support and CDC on couples’ dyadic management of T2DM and their well-being in daily life. Using AAMC will make a key contribution with regard to objective operationalizations of visible and invisible support, CDC, physical activity, and well-being. Findings will provide a sound basis for theory- and evidence-based development of dyadic interventions to change health behavior in the context of couple’s dyadic illness management. Challenges to this multimodal sensor approach and its feasibility aspects are discussed.

**International Registered Report Identifier (IRRID):**

PRR1-10.2196/13685

## Introduction

Type II diabetes mellitus (T2DM) is a common chronic disease of the endocrine system in which the pancreas no longer produces enough insulin to metabolize blood glucose or the body becomes less sensitive to insulin (ie, insulin resistance) [1]. Symptoms include numbness in the hands or feet, excessive thirst and urination, and nausea [1,2]. Worldwide, 366 million people suffer from T2DM, which corresponds to 8.3% of the world population [1]. More than 1 in 4 adults aged 65 years and older are estimated to have T2DM in the US population, resulting in 9.4% of the US population [3]. If the trend continues, 1 of 3 US citizens will have T2DM by 2050 [4]. In Switzerland, almost 500,000 people suffer from T2DM, which is approximately 4.9% of the male Swiss population and 4.2% of the female Swiss population [1,5]. The prevalence rates raise with increasing age: 15.3% of males and 11.3% of females aged older than 75 years are diagnosed with T2DM [5]. To manage blood glucose levels and reduce the risk of diabetes-related complications (eg, cardiovascular diseases, vision loss, and amputations), patients need to follow medical recommendations for healthy eating, physical activity, and medication adherence in their everyday life [3]. Most T2DM patients take oral antidiabetic drugs [6]. Diabetes management is, hence, a very complex endeavor and requires lifelong commitment and modification of one’s personal lifestyle [4,7]. Evidence suggests that for married adults, illness management is mainly shared with their spouses [8,9]. Spousal involvement in patients’ diabetes management may involve social support [10] and common dyadic coping (CDC) [11,12]. Until now, it is unknown which of these concepts (ie, visible support, invisible support, and CDC) displays the most beneficial effects on the diabetes management in romantic couple’s everyday life when considered together. Therefore, the aim of this study is to systematically investigate the effects of social support and CDC on health behaviors involved in diabetes management (eg, physical activity, healthy diet, and medication adherence) and well-being using a novel ambulatory assessment application for smartphones for the open-source behavioral intervention platform MobileCoach (AAMC [13]) [14,15] that allows the objective evaluation of the core study constructs and outcomes in romantic couples’ everyday lives.

### Received Social Support, Health Behaviors, and Well-Being

*Social support* describes the provision of resources intended to benefit a receiver’s ability to cope in times of need [16]. The most prominent functions of support are emotional (eg, comforting) or instrumental (eg, practical assistance) [17]. Recipients’ reports of support received can be distinguished from providers’ reports of support given [18]. The perspectives of receiver and provider do not necessarily closely correspond, even when support receipt and provision concern the same reported support from 2 different persons [19]. There is a growing body of research reporting positive associations between received support and health behaviors, such as dieting or increased physical activity in the general population (eg, studies by Scholz et al [20], Darlow and Xu [21], Courneya et al [22], and Molloy et al [23]). In the context of chronic illness, spouses or partners are mostly the main sources of support [24]. Support from spouses has been associated with positive outcomes for patients with chronic health conditions, such as healthier eating habits among diabetic patients [25], increased health behavior (eg, eating a healthy diet, engaging in physical activity, and avoiding highly stressful situations) in cardiovascular patients [26], and decreased risk behaviors in HIV patients [27]. In this research area, only few studies have examined social support and health behaviors on a day-to-day basis (for an exception, refer the studies by Khan et al [7], Iida et al [28], and Stephens et al [29]). Diabetes symptoms are very sensitive to lifestyle behaviors and fluctuate daily [30]. Therefore, T2DM offers an ideal context for investigating associations between partner involvement, well-being, and daily illness management. For example, focusing on daily processes revealed that relative to the previous day, spouses’ diet-related support was associated with increases in patients’ adherence to diet [29] or that daily spousal support was positively associated with patients’ daily physical activity [7]. Therefore, it seems that social support provided by a partner is especially beneficial for T2DM patients’ illness management.

Research on received social support and coping with stress has shown that being in a supportive relationship can buffer physical and psychological effects of illness-related stress in general [31] and regarding T2DM in particular [28]. In line with this finding, Stephens et al [29] found that spouses’ diet-related support was associated with decreases in diabetes-specific distress of T2DM patients. Contrary to this result, studies on received support and indicators of well-being oftentimes result in negative associations between received support and well-being (eg, studies by Bolger et al [32], Gleason et al [33], and Seidman et al [34]). As a consequence, Bolger et al [32] introduced the dyadic concept of *invisible social support*, which is assumed to provide the benefits of support receipt without including potential costs. According to Bolger et al [32], support is invisible to recipients when the supportive acts occur outside of their awareness (ie, one partner takes care of unexpected housework without telling the other) or the recipient may be aware of the act but may not code it as support (ie, one partner purposefully gives advice in an indirect way so as not to draw attention to the recipient’s distress or his/her inability to deal with the stressful situation). To date, the few studies that have investigated invisible support in prospective diary designs (eg, studies by Bolger et al [32], Shrout et al [35], Maisel and Gable [36], and Biehle and Mickelson [37]), observational studies (eg, studies by Howland and Simpson [38] and Girmeet al [39]), and experimental designs (eg, studies by Bolger and Amarel [40] and Kirsch and Lehman [41]) have yielded support for beneficial effects on well-being and encourage further research.

The applicability of invisible support for health outcomes has been discussed (eg, studies by Kirsch and Lehman [41], Taylor et al [42], and Westmaas et al [43]). Until now, only 2 daily diary studies focused on behavioral responses to invisible support [44,45]. In single-smoker couples, higher invisible support was associated with less distress, but with more daily cigarettes smoked after a self-set quit date [44]. In dual-smoker couples only for men invisible support was associated with less distress, but not with more smoking after a joint self-set quit date [44]. Thus, based on these first 2 studies, it seems as if invisible support can also counteract the negative effects of visible support on well-being in the context of health behavior change. However, the results with the health behavior itself emphasize the need for further understanding the interplay between invisible support and health behaviors in different health contexts. Until now, no study was conducted in the context of chronic illness, and the relevance of invisible support in T2DM with regard to diabetes-related health behaviors and well-being needs yet to be demonstrated.

Most studies so far assessed invisible support by calculating a composite score from 2 self-reports (target person and partner). On the one hand, researchers assessed invisible support dichotomously (eg, studies by Bolger et al [32] and Shrout et al [35]). In this approach, invisible support was coded when the target person reported no receipt of support, but the support provider reported provision of support. On the other hand, researchers calculated continuous invisible support by subtracting received support reported by the target person from provided support reported by partners (eg, studies by Biehle and Mickelson [37] and Lüscher et al [44,45]). Instances in which the recipient reported receiving more than the provider reported giving were collapsed to zero. However, these measures are often criticized not only because invisible support is based on self-report but also because it is merely calculated and thus potentially only a hypothetical construct. Therefore, studies in which invisible support is measured directly, observed, and coded in everyday life as has already been done in the laboratory (eg, studies by Howland and Simpson [38] and Girme [39]) are strongly needed.

### Common Dyadic Coping, Well-Being, and Health Behavior

The usual approach followed by social support researchers is unidirectional in that support provision of one individual (usually the healthy one), and support receipt of another individual (usually the unhealthy one) is in focus. Invisible support already introduces a dyadic conceptualization in that both perspectives of partners are usually needed for the assessment of invisible support. CDC, however, goes beyond this dyadic implementation of still individual behaviors in that CDC explicitly focuses on the joint, dyadic efforts a couple undertakes to overcome challenges and problems (eg, a study by Bodenmann [11]). There are different approaches to CDC, but all these conceptualizations share the view that CDC involves a *we* approach with regard to stress (eg, studies by Bodenmann [11], Acitelli and Badr [46], and Kayser et al [47]) and health behaviors (eg, studies by Johnson et al [48], Lewis et al [49], and Lipkus et al [50]). Thus, CDC explicitly refers to the couple’s perspective and comprises joint efforts to cope with a stressor at the couple’s level. In the context of diabetes management, this could mean that the couple solves all issues related to the patient’s change of behavior together. Overall, research on CDC consistently results in positive associations with well-being and relationship quality (eg, studies by Bodenmann et al [51] and Traa et al [52]). Results of the association between CDC and health behavior, however, are mixed. Some studies report positive associations between CDC and health behaviors (eg, studies by Johnson et al [48] and Rohrbaugh et al [53]; for an experimental approach, refer to the study by Lipkus et al [50]). Along similar lines, within the context of T2DM, Seidel et al [9] showed that shared expectations regarding partner's diet-related involvement were positively associated with diet adherence in male patients. Benefits of a dyadic approach of patient’s and partner’s illness representation of T2DM were also documented by Dimitraki and Karademas [54]. Yet, another experimental study in the context of diabetes management does not support the hypothesis that CDC interventions are superior to individual interventions, albeit the dyadic group proved to be better than the control group (eg, studies by Trief [55]).

There are several ways in which CDC can be measured. The usual way of assessing CDC outside the laboratory is through self-report (eg, using the respective subscale of the Dyadic Coping Inventory) [56]. At the same time, a coding system for laboratory observations exists [56]. Another objective alternative is the assessment of we-talk by counting the use of first-person plural pronouns (eg, a study by Rohrbaugh et al [57]). Indeed, in a study by Rohrbaugh et al [57] that distinguished between we-talk and self-reported CDC, authors found that it was the objectively measured we-talk, but not the self-reported communal coping that predicted heart failure symptoms and general health. Thus, this study sets out to measure CDC with 2 assessment methods combined: self-report and we-talk on a daily basis in the context of dyadic diabetes management.

### Subjective and Objective Ambulatory Assessment by Mobile Phone Apps

With regard to an in-situ assessment of the theoretical constructs of this study (ie, visible support, invisible support, and CDC), mobile phone applications are a powerful tool for several reasons [58-64]: the widespread use of smartphones and smartwatches with various sensors and touch-based graphical user interfaces makes sophisticated assessments of theoretical constructs appealing and widely applicable. Second, the combination of sensor data of smartphones and smartwatches (eg, from the global positioning system sensor or microphone) and their proximity to their owners offers the ability to detect useful contextual information (eg, the geographic position or mood of the owner). Third, mobile phone applications are scalable, cost-effective, have low entry barriers, and are applicable to different target populations. Finally, mobile phone applications reach people in their everyday life and with an immediacy that observations using conventional research methods do not have. In recent years, researchers have increasingly begun to use smartphones and smartwatches as platforms for the assessment of health behavior. However, although there exist various mobile phone applications to monitor behaviors and outcomes related to diabetes management in general (eg, studies by Sun et al [65], Schembre et al [66], and Wang et al [67]) and individual facets of it such as physical activity (eg, studies by Joosen et al [68] and Bort-Roig et al [69]), nutrition behavior (eg, studies by Celis-Morales et al [70], Turner‐McGrievy et al [71], and Hassannejad et al [72]), well-being (eg, studies by Dubad et al [73] and Servia-Rodríguez et al [74]) or conflict in couples (eg, a study by Timmons et al [75]), mobile phone applications that use objective sensor data in combination with self-reports are so far not used for the ambulatory assessment of social support and CDC.

A prominent example of a mobile phone application for the unobtrusive assessment of natural language and communication in real life is the electronically activated recorder (EAR) [76]. The EAR collects audio snippets at random times that can be coded with regard to the content of interest for the respective studies. For example, there are applications of the EAR with regard to social support provision in couples coping with breast cancer (eg, a study by Robbins et al [77]). The focus of the EAR, however, is on auditory observation only. Novel sensor–based approaches of affect recognition (eg, studies by Betella and Verschure [78], Maass et al [79], Venkatesh et al [80], van der Heijden [81], Chapaneri and Jayaswal [82], Revathy et al [83], Koolagudi and Rao [84], Heron and Smyth [85], Spanier [86], and Diener et al [87]) can be used in combination with appropriate self-report scales such as the affective slider [78] to better understand outcome parameters of well-being in the context of diabetes management.

A number of open questions remain from the current literature on visible and invisible social support, CDC, and its ambulatory assessment by mobile phone applications that will be addressed in this study. First, no study has examined the 3 concepts of visible and invisible social support and CDC in 1 study. Thus, the unique contributions of these constructs on health behaviors and well-being have not yet been examined. In particular, with regard to health behavior change, there is insufficient knowledge on the effects of invisible social support. The second open question in the current literature on invisible support and CDC concerns the assessment in everyday life. Measures of invisible support are often criticized for being not only merely based on self-report as measures of visible support but also merely calculated from independent reports of receipt and provision of support. Thus, invisible support is potentially only a hypothetical construct, and studies in which invisible support is measured directly, observed, and coded in everyday life are strongly needed. With regard to CDC, most studies so far focused on either cross-sectional associations or longer-term associations between CDC and health behavior. Associations in everyday life using an ambulatory assessment approach have been neglected so far. Given the assumed importance of invisible support and CDC for health behaviors involved in diabetes management and well-being in T2DM patients and their partners, it is of key importance to assess these constructs in a reliable and valid objective way in everyday life to further our knowledge. Third, it is still to be investigated how to design an ambulatory assessment application for the purpose of this study, which is not only accepted by study participants in their everyday situations [79-81] but also delivers high-quality data streams that are good enough or even comparable with distinct devices (eg, high-quality microphone for affect recognition from speech in the laboratory). Fourth, available speech databases and latest research on affect recognition from speech employ usually role-taking actors and thus lack natural settings and, with it, external validity (eg, studies by Chapaneri and Jayaswal [82], Revathy et al [83], and Koolagudi and Rao [84]). Finally, multimodal approaches to affect recognition are promising, but existing research is sparse, and consistent results and approaches are still to be explored [75]. This study will address all these open questions and limitations of previous research.

### Aims of This Study

The aims of this study are to address the open questions outlined above. The first aim is to examine the impact of visible and invisible support and CDC on couple’s dyadic management of T2DM (ie, health behaviors) and well-being. The second aim is to use an improved assessment approach of visible and invisible support and CDC in everyday life, which is based on observational and self-report data in situ instead of self-report only. The third aim is closely related to the first and second aims: that is, to develop an AAMC that allows to record both multimodal sensor data streams and self-reports related to the study’s core constructs in situ to better understand visible and invisible social support and CDC and the relationship between the recorded sensor data and psychological self-reports. To better understand outcome parameters of well-being in the context of diabetes management, the fourth aim is to use a multimodal affect recognition approach (ie, speech, facial expression, and heart rate variability) in combination with self-report scales.

The first aim of the study will be examined in daily life as well as in an observational setting in the laboratory, whereas the second and fourth aims refer to the experience sampling phase of this study. The results of the third aim are finally used to support the first two and the fourth aims by capturing and assessing the core study constructs by self-reports and objective measures during the experience sampling phase. The following research questions result from these study objectives: (1) What are the unique contributions of visible and invisible social support and CDC for diabetes patients’ health-related behaviors involved in diabetes management (physical activity, diet adherence, and medication adherence)? (2) What are the unique contributions of visible and invisible social support and CDC for indicators of well-being derived from multimodal data sources and self-reports captured by AAMC in diabetes patients and their partners?

## Methods

### Study Design

**Figure 1:**
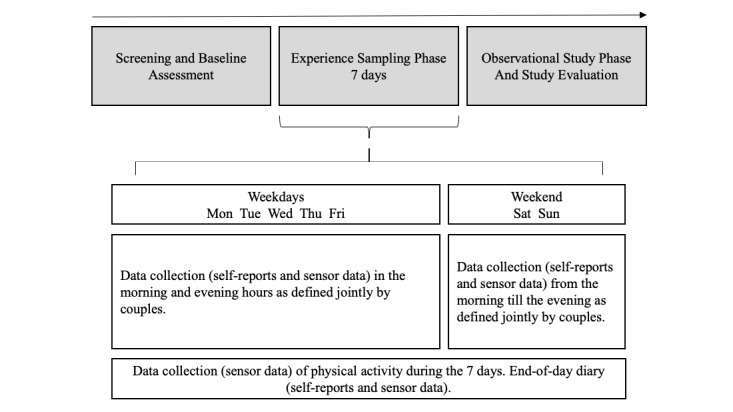
Study design.

This study protocol describes the design of the Dyadic Management of Diabetes (DyMand) study, funded by the Swiss National Science Foundation (CR12I1_166348/1). The study was approved by the cantonal ethic committee of the Canton of Zurich, Switzerland (Req-2017_00430). To address the aims mentioned above, this study comprises an intensive longitudinal design with 2 phases of data collection. The first phase is an experience sampling phase in romantic couple’s everyday life (7 days) following an ecological momentary assessment (EMA) approach [85]; the second phase is an observational study phase in the laboratory. Figure 1 shows the study design.

### Sample and Recruitment

The target population of this study are 180 patients with T2DM and their romantic partners. Inclusion criteria are the medical diagnosis of T2DM of the target person with prescribed oral antidiabetic drugs and having a partner of the opposite sex without diabetes or a psychological disorder who is also willing to participate in the study. The participating couples should be in a close, committed relationship for at least 1 year and living together in 1 household for at least 6 months. Exclusion criteria include T2DM treatment with insulin, inpatient treatment, shift work of one or both partners, and insufficient knowledge of the German language.

Recruitment of patients will take place in several hospitals in Switzerland. Moreover, couples will be recruited by means of flyers in medical clinics, hospitals, private practices, pharmacies, and Schweizerische Diabetes-Gesellschaft (Swiss Diabetes Society), information provided to physicians who will inform patients actively about the study, and Web-based forums for diabetes patients and diabetes-related websites. Furthermore, we plan to invite patients through radio and television formats and health magazines.

### Detailed Description of the Study

Objective and self-report data from both partners are collected and assessed throughout the study to allow focusing on effects of both partners.

#### Screening and Baseline Assessment

Interested couples will be asked to consent to and then complete a Web-based questionnaire to screen inclusion and exclusion criteria and assess sociodemographic information. Moreover, they will receive first information about the study. Eligible couples are then invited to the laboratory of the Applied Social and Health Psychology Group at the University of Zurich for a baseline assessment. During this session, both partners will receive comprehensive information about the study, sign the informed consent form, and fill in a Web-based questionnaire capturing all constructs of interest at baseline that are not assessed on a daily basis, but will later serve as control variables. Control variables are duration of relationship, duration of living together, duration of T2DM illness, severity of illness, oral antidiabetic drugs prescribed, illness symptoms, physician’s recommendations for diabetes management, and relationship quality of both partners with different dimensions such as consensus, cohesion, and satisfaction (Dyadic Adjustment Scale) [86] as well as life satisfaction of both partners (Satisfaction With Life Scale) [87], technology anxiety [88], familiarity with mobile phone text messaging applications [89], and experience with and usage of smartphones, smartwatches, and step counting devices [80,88]. They are handed over the study smartphones (1 for each partner; Nokia 6.1, 2018, Android operating system 9.0) and the study smartwatches (1 for each partner; Polar M600, Google Wear operating system 2.3) and are instructed how to use the newly developed AAMC, which will collect the multimodal sensor data and capture self-reports of both partners of the participating couples. Moreover, during the 7-day experience sampling phase, all participants (target persons and partners) wear triaxial accelerometers at the hip (GT3X+ monitor devices; ActiGraph).

#### Experience Sampling Phase

The experience sampling sequence starts for all participating couples on the following Monday after baseline assessment and ends the following Sunday night to have the same sequence of days for all participants. Both partners are sent an automatically generated text message to their own mobile phones on Sunday evening and again on Monday morning reminding them to put on the study smartphones, wear the study smartwatches, and accelerometers to use AAMC directly after getting up. Participants are instructed to have all devices with them every day for the 7 days from getting up until going to bed (for a similar procedure during weekends, refer to the studies by Robbins et al [90] and Helgeson et al [91]).

The AAMC was developed as an open-source extension of the existing MobileCoach platform [13] by applying design science research. The AAMC consists of a smartphone app, a smartwatch app, and server system built on top of MobileCoach [92]. MobileCoach is a server-client system that allows both the collection of sensor and self-report data (eg, for EMA studies or for health monitoring purposes) and the delivery of health interventions [14,15]. On the server side, the data collection and intervention logic are defined (eg, when to collect which information), whereas short message service text messages and mobile phone applications for Apple’s iOS and Google’s Android operating systems are used to actually collect that data and deliver the interventions. MobileCoach follows the talk-and-tools paradigm [93]; that is, it provides *tools* to collect data and intervene on the one hand (eg integration of Web-based surveys or the provision of health literacy video clips) and, on the other hand, to interact with subjects through a digital coach, also known as chatbot or conversational agent [94], the *talk* component. In this study, the digital AAMC coaches PIA (interacting with the partner with diabetes) and PETE (interacting with the partner without diabetes) have been designed to talk to the subjects during the experience sampling phase with the help of a chat-based interface with predefined answer options as successfully carried out in previous work (eg, studies by Kowatsch et al [15,95]).

Design science is a methodology-guided iterative development of information systems (ISs) and rigorous evaluation of IS deployments [96,97]. During the build phases, mobile services were implemented (1) that record multimodal sensor data related to the study’s core constructs through the study smartwatch, study smartphone, and a dedicated physical activity device worn on the hip and (2) that record self-report data of the study’s core constructs through the smartphone.

The experience sampling is conducted as follows. A 5-min recording of audio, heart rate, gyroscope, ambient light, and accelerometer data through the study smartwatch is triggered when the partners are close to each other and when an acoustic signal of *no silence* was detected. The closeness of the partners is measured by the Bluetooth’s signal strength of the 2 smartwatches. Directly after the 5-min recording, subjects are notified through an acoustic signal on the study smartphone and vibration on the study smartwatch to fill out a brief questionnaire (details are provided below) by the AAMC digital coaches PIA and PETE from within the chat-based user interface of the AAMC. If subjects do not start to fill out the brief questionnaire within 2 min, another acoustic signal and vibration are triggered. If then, within 2 min, still no response was detected, filling out the self-report is not possible anymore. During the process of filling out the questionnaire, a short 3-second video clip of the participant’s facial expression is recorded with the front-facing camera of the study smartphone. The following 2 constraints were added to balance the number of sensors and self-report recordings and the burden of participants: (1) at least one recording of 5 min is conducted per hour; that is, if the recording is not triggered for 45 min as described above (ie, by the Bluetooth and acoustic signal), a backup recording is done in the last 15 min of that hour; and (2) the start of 2 recordings has to be at least 20 min apart from each other. Finally, closeness between the 2 partners is measured in a regular time interval with the help of the Bluetooth’s signal strength of the 2 smartwatches during the relevant recording hours.

The relevant recording hours for this experience sampling are the hours in the morning and evening during the weekdays, that is, experience sampling days 1 to 5. These hours are defined by the couples during the onboarding process at the baseline assessment and can be set from 4 am, 5 am, 6 am, 7 am, 8 am, and 9 am to 6 am, 7 am, 8 am, 9 am, 10 am, and 11 am for the morning hours and from 4 pm, 5 pm, 6 pm, and 7 pm to 9 pm, 10 pm, and 11 pm for the evening hours. During the weekend, that is, experience sampling days 6 and 7, only the early morning hours and late evening hours are set (eg, from 6 am to 10 pm). With this procedure, privacy aspects are addressed by primarily focusing on situations, in which the couples will be spending time together and thus to reduce the number of audio recordings during the day of weekdays when chances are higher that subjects are working, moving around in public places, or visit or are visited by friends.

The brief questionnaire on the smartphone assesses patients’ received and partners’ provided support, CDC, and affect by valence and arousal for the past 5 min. Patients report if they received support from their partners (yes/no) and partners report if they provided support to their partners (yes/no) in the last 5 min. If the answer is yes, they are asked in what domain they received/provided support (physical activity, diet adherence, medication adherence, and other). Moreover, patients report their perception of CDC with the inclusion of other in the self-scale and the structure of interpersonal closeness [98]. Items for the partners will be directly parallel but referring to “your partner’s diabetes condition.” In addition, the speech recordings will be transcribed and coded or coded directly from the audio files with regard to the receipt and provision of social support from both partners [90]. Moreover, using the system for coding dyadic coping (SEDC) [56] and the coding system of we-talk [53], the audio files will also be coded with regard to CDC. In doing so, invisible support will be identified when provided support by the partner is coded from the audio recordings, but the recipient does not report receipt of support in the subsequent questionnaire. With this method, we will be able to overcome the above-mentioned methodological problems of assessing invisible support in people’s everyday life. At the same time, reports of receipt of support are the indicators of visible support. Using these different assessment methods also allows for testing the unique effects of the 3 constructs: visible support, invisible support, and CDC without running into problems of completely shared method variance.

Furthermore, the self-reported valence and arousal dimensions of affect as experienced in the last 5 min are assessed with the affective slider [78]. This affect measure is used to assess its relationship with the multimodal sensor data to derive a novel digital biomarker for affect based on voice features (eg, prosody), heart rate, ambient light, gyroscope, accelerometer data, and facial expressions [75,99]. Both self-reported affect instrument and the multimodal sensor data linked to affect will help to deepen our understanding of the outcome well-being.

On every day during the 7-day experience sampling phase, participants will also be asked by PIA and PETE from within the chat-based user interface of AAMC to complete a short end-of-day diary with more comprehensive questions on social support and CDC, healthy eating, medication adherence, and well-being of both partners to cover also the times of the day that are not captured by the random audio recordings and subsequent brief self-reports. Measures in the end-of-day diary are adapted from the studies by Bolger et al [32] received and provided *social support* is assessed by asking patients “Today, I received emotional/instrumental support from my partner” and by asking partners “Today, I provided emotional/instrumental support to my partner.” Emotional and instrumental support will be briefly defined for participants. Moreover, patients will report their perception of CDC with the items (1) “When you think about problems related to your diabetes condition today, to what extent did you view those as ‘our problem’ (shared by you and your partner equally) or mainly your own problem?” with a bipolar response scale from 1= *today completely my own problem* to 6= *today always our problem* and (2) “When today a problem related to your diabetes condition arose, to what extent did you and your partner work together to solve it?” with a response scale from 1= *today not at all* to 6= *today very much*. Both items are adapted from Rohrbaugh et al [57] to a daily basis. Items for the partners will be directly parallel but referring to “your partner’s diabetes condition.” For *dietary adherence*, patients report the extent to which they had followed a recommended diet. *Medication adherence* is assessed with the Medication Adherence Rating Scale [100] adapted to a daily basis. Thereof, for dietary adherence and medication adherence, a dichotomous measure (adherence to recommendations yes-no) results. Finally, psychological *well-being* is assessed with the short form of the Positive and Negative Affect Schedule [101] and with the affective slider [78] on a daily basis of both partners. During this end-of-day diary and similar to the experience sampling procedure, a short 3-second video clip of the participant’s facial expression is recorded with the front-facing camera of the study smartphone. This recording together with the additional multimodal sensor data collected over the course of the day will be used to investigate the relationship between self-reported measures of affect and well-being and the multimodal sensor data [99].

In addition to the experience sampling and the end-of-day diary, physical activity is measured continuously throughout the 7 study days with the study smartwatch and a triaxial accelerometer worn on the hip (GT3X+ monitor devices). To parallel recommendations for physical activity [102], a measure of the minutes of moderate-to-vigorous physical activity will be created by summing the minutes of moderate exercise and vigorous exercise from the accelerometer data. By doing so, a dichotomous measure (adherence to recommendations yes-no) results. Furthermore, the ActiGraph is used to validate the physical activity data of the study smartwatch with the overall objective to assess the need of a device that measures physical activity in addition to the accelerometers integrated into the smartwatches. In the best case, consistent results among the different devices would lead to a removal of the dedicated device and thus to decrease the burden of subjects in future EMA studies or health interventions.

#### Observational Study Phase

After the 7-day sequence, participants will return to the laboratory to hand in the study smartphones, smartwatches, and accelerometers. Moreover, couples will then participate in the second part of the study, the observational study, and complete a final questionnaire on the AAMC on their study smartphones. The observational study examines visible and invisible support and CDC by analyzing couple’s videotaped discussion about diabetes-related concerns during a 10-min discussion.

Using the same procedure as was used by Dagan et al [103] and Badr et al [104], T2DM patients and their partners will be asked to list their T2DM-related and illness management–related concerns and select one that is causing them considerable distress. Next, they will be invited to discuss the issue with their partner for about 10 min in a videotaped session. The task will be guided by a psychologist who will leave the room during the discussion. The underlying idea is that the discussions will capture how couples talk about T2DM-related concerns. During the discussion, each partner will wear a smartwatch as it collects various sensor data similar to that in the ambulatory setting for the experience sampling phase. Following the discussion, both partners will report their perception of the discussion, and both partners will rate the discussion in terms of the degree to which it has been typical of their discussions at home, how helpful it was, and how it made them feel. Furthermore, both partners will complete measures on how much they felt supported and how much they were themselves providing support. This allows the assessment of invisible support by coding provision of support and self-reported receipt of support [38,39]. Also, they will each complete the Affective Slider self-report, assessing the valence and arousal dimensions of their affect over the last 10 min of the discussion. The videotaped discussions are subsequently coded by trained observers for visible and invisible support transactions and CDC. For this, a codebook will be developed based on previously published work for visible and invisible support [38,39,104,105]. We will use the SEDC [56] for coding CDC. Moreover, 2 trained blinded coders, showing high interrater reliability (kappa) after training, will review the videotaped discussions for the support provided (observer-rated support) and dyadic coping strategies independently. This procedure will not only consider both perspectives of social support of a couple but also an observer perspective as suggested by Dunkel-Schetter et al [19].

Finally, couples will complete separately from each other a Web-based survey on technology acceptance constructs with regard to AAMC such as perceptions of enjoyment, ease of use, usefulness, and the intention to interact with the digital AAMC coaches PIA and PETE [80,94,106-109]. In addition, consistent with previous research on technology acceptance, 7-point Likert scales ranging from strongly disagree (1) to strongly agree (7) will be used. To assess the attachment bond of the participants with the digital coaches PIA and PETE and also the shared understanding between them and subjects with respect to the EMA goals and tasks, a short version of the working alliance inventory for technology is adopted from previous work [110-113]. In particular, we will use the Session Alliance Inventory by Falkenström and Hatcher [113] with 6 items because of the short duration of the EMA study with a 6-point response scale ranging from not at all (1) to completely (6). Finally, subjects are asked to indicate potential improvements related to the AAMC. All the couples will receive a compensation of CHF 100 for their time and travel expenses.

### Statistical Analysis

The main research aims of this study refer to between-person associations of visible support, invisible support, CDC, patient’s diabetes-related health behavior, and well-being of both partners using a dyadic approach to account for the interdependence among couple members using a 2-level statistical model for distinguishable dyads as indicated for patient-partner dyads [114]. The main analyses will be correlations and multiple regression analyses, which will be performed in SPSS and R. For the diabetes-related health behaviors, which relate to physical activity, diet, and medication adherence, we will generate a daily composite score, indicating the meeting of the recommendations for all 3 behaviors together ranging from 0 (meeting none of the recommendations) to 3 (meeting all of them). Moreover, we will also be able to analyze associations between predictors and the different behaviors assessed continuously in separate analyses. The idea of the composite score, however, takes into account that the real-life assessment method we chose to capture invisible and visible support and CDC using objective measures might result in highly ecologically valid and reliable measurements, but potentially in a rather low frequency of these predictors for the different diabetes-related health behaviors. With regard to well-being, we will consider the affective valence and arousal assessed during the experience sampling sequences or the mean scores of positive and negative affect from the end-of-day assessments. On a more exploratory level, we will also analyze day-to-day within-person and within-couple associations in further analyses using multilevel modeling. But because of the novelty of our approach and the rather short time frame of 7 days, this will not be the main focus.

To assess the relationship between the sensor data and self-reports, machine learning is applied, which is carried out in several steps. First, preprocessing of the raw sensor data involves feature extraction, feature scaling, feature selection, and dimensionality reduction. The resulting features derived from sensor data will be tested in machine learning models to predict self-reported affect and well-being. Second, the data will be split into training and test datasets to assess how derived algorithms generalize to new data [115]. The training dataset will also be split into subsets, where a k-fold cross-validation will be applied. The performance of the resulting model will then be evaluated using the test data set. This procedure will be repeated for various learning algorithms (eg, random forest, support vector machines, naive Bayes, recurrent neural networks, and feedforward neural networks). After comparing the performance across algorithms, the best overall model will be selected. We expect to produce a model that efficiently predicts affect and well-being using a multimodal compared with a unimodal approach as outlined in previous work on affective computing [99].

### Power Analysis and Sample Size

The sample size was calculated based on Cohen [116] and using the G*Power program [117] to secure adequate power for the primary outcomes. There are no meta-analyses available for the associations between visible support, invisible support, and CDC with health behaviors and indicators of well-being. Moreover, studies reporting results from diary studies use unstandardized effects. Thus, we base our power calculation on data from previous studies on these associations, but we are aware that the data basis is somewhat unsatisfactory to make strong conclusions about the expected effect sizes. Previous studies reported varying effect sizes for visible received support on health behavior (eg, r=0.29-0.34) [20,23]. There are only 2 studies so far that examined the association between invisible support and health behavior (eg, smoking) [44,45]. In these 2 studies, however, no standardized effect sizes are available because of the focus of within-person effects. In the case of visible and invisible social support and their relations to well-being, we draw on the experimental evidence available [40]. In a series of 3 experiments, Bolger and Amarel [40] demonstrated that visible support compared with a no-support control group was related to increases in distress (*d*=0.27 and *d*=0.66, ranging from small to medium effect sizes). Invisible support, in contrast, leads to lower distress (*d*=−0.63 and *d*=−1.09, indicating medium to large effect sizes). The difference between the effects of invisible and visible support resulted in a large effect (*d*=−1.09). Thus, medium effect sizes for the associations between visible and invisible support and indicators of well-being will be expected. In the case of CDC, previous studies report effect sizes with behavior of *r*=0.20 with exercise adherence, *r*=0.20 with dietary adherence in a sample of diabetes patients [48]. Moreover, in the domain of well-being, effect sizes range from *r*=0.20 to *r*=0.33 (eg, a study by Badr et al [118]).

As we will not only address bivariate associations between our predictors and criteria but aim at comparing effects between our predictors, our calculation of the power is based on a 2 dependent Pearson r’s analysis with a common index. To the best of our knowledge, no published data on the intercorrelations of visible support, invisible support, and CDC are available. Data from our own research with smoking-nonsmoking couples (excluding CDC) resulted in an association of *r*=−0.25 for visible and invisible support. To detect a significant association with a continuous outcome at *P*<.05 with a power of 1−beta=0.90, and assuming a small effect size of *r*=0.2, for health behavior and visible support, *r*=−0.2, for health behavior and invisible support and an intercorrelation of *r*=−0.25 between visible and invisible support, the required sample size is 164. Because effect sizes for CDC tended to be higher, the study will be powered for the smaller effect sizes of support. Previous studies applying intensive longitudinal designs resulted in very low rates of dropout even across a longer period (ie, ≤10%) [119-121]. This can be explained with a high commitment in couples willing to participate in these kinds of studies. On the basis of this experience, we will add 10% to the calculated sample size to account for potential dropout. This results in a total required number of 180 couples.

Analyses of the relationship between multimodal sensor data and self-reported data on affect and well-being are conducted with the help of machine learning. We estimate that in the best case, we will have an average of 10 completed self-reports per day for all 7 days resulting in 25,200 samples (360 individuals×10 self-reports×7 days). It is highly likely to have missing data if subjects do not complete the self-reports. Therefore, in a worst-case scenario with only 1 self-report completed per day and a corresponding 90% missing data, there will be 2520 observations left to use to train the machine learning algorithm. Owing to the fact that there exists no widely accepted sample size calculation method for machine learning approaches, the sample size of 360 individuals with up to multiple measurements on 7 consecutive days (eg, at least 2520 observations only from the end-of-day diary) lies above related work (eg, studies by Wahle et al [60] and Timmons et al [75]) and thus is assumed to be adequate for the purpose of this study.

### Data

In line with the open research data initiative of the Swiss National Science Foundation, the anonymized data of the study will be made public in a noncommercial database for replication purposes of the analyses, additional data analyses by any third parties (eg, other research groups), and quality control purposes, given that there are no ethical or legal restrictions.

## Results

The AAMC was designed and built until the end of 2018 and internally tested in March 2019. In May 2019, the enrollment of the pilot phase began. The data collection of the DyMand study will begin in September 2019, and analysis and presentation of results will be available in 2021.

## Discussion

The impact of social support and CDC on health behavior change and well-being has attracted researchers’ interests for some time. Researchers found that social support and CDC are associated with benefits for health behaviors and well-being (eg, studies by Scholz et al [20], Stephens et al [29], Johnson et al [48], Bodenmann et al [51], Rohrbaugh et al [53], and Uchino et al [122]) but often also with costs and harmful consequences (eg, studies by Bolger et al [32], Gleason et al [33], Seidman et al [34], Westmaas [43], and Trief [55]). For further research and practical reasons, it is crucial to identify which types of social support and CDC are beneficial and which are potentially harmful for health behaviors and indicators of well-being. Diabetes management is an ideal field to tackle this task. T2DM is a widespread disease and can be treated and managed by following a healthy meal plan, regular physical activity, and taking medications to lower blood glucose levels. Thus, patients’ education and self-care practices are important aspects of T2DM management that help patients to stay healthy. The social environment has been found to be highly influential in the illness management process [10,11] although effects of social support and CDC on health behavior change and well-being in the context of T2DM management are not yet well understood. This study is the first to systematically investigate couples’ dyadic illness management by investigating visible and invisible support and CDC in T2DM patients and their partners in daily life by applying an experience sampling approach and an observational approach on the basis of the new open-source behavioral intervention platform MobileCoach [14,15].

This combined experience sampling and observational approach is highly relevant for the following reasons: analyzing visible and invisible support and CDC in daily life has thus far not been done with a focus on a dyadic perspective by considering T2DM patients and their partners. Furthermore, so far it is unknown which of these concepts displays the most beneficial associations with well-being and health behavior when considered together. Moreover, measurement of these constructs in people’s everyday life is by self-report only. Current technical developments allow using alternative, more objective operationalizations of these constructs and solve problems linked to self-report instruments. However, with the method applied in this study, we will not be able to capture all forms of invisible social support. For example, all kinds of nonverbal support behaviors, such as hugging as a form of emotional support, will not be detected. Nonetheless, we consider this assessment method innovative and advantageous to mere self-report measures of invisible support. Furthermore, this study will not only substantially advance the knowledge in the area of couple’s dyadic management of T2DM but also on the important question of which supportive or coping acts are positively related to health behavior change and well-being. Therefore, this will provide a sound basis for the development of theory-based and evidence-based dyadic interventions to change health behavior.

Furthermore, the technical contribution of this project, that is, the enhancements of the MobileCoach platform with its newly developed modules (AAMC) and its capabilities to capture subjective self-report data and objectively physical activity and affect through a multimodal sensor fusion approach, will be made open source under the research and industry-friendly Apache 2 license [123]. Thus, we expect high adoption rates and further developments of the AAMC by public institutions, business organizations, and interdisciplinary research teams in the field of ISs, computer science, health psychology, and behavioral medicine. The AAMC is generic in that it may not only be suitable for diabetes management but also for related diseases such as obesity, hypertension, or mental health disorders in which self-reports, physical activity, and affect detection take over a key role for diagnosis and health intervention designs. Moreover, given a sufficient degree of classification accuracy in using sensed and EMA data to predict affect and well-being, these models could then be used to help reach vulnerable individuals early and provide appropriate just-in-time adaptive interventions [124], respectively.

There are several issues that might raise questions on the feasibility of this study. First, speech recordings have repeatedly been questioned with regard to research ethics because it might happen that people not involved in the research study are being recorded too or that participants’ privacy is endangered. With regard to protecting participants’ privacy, for example, all participants have the opportunity to listen to their audio files and request their deletion without giving any reason and without anyone else listening to them as already applied by Robbins et al [77,90]. Moreover, with regard to the recording of other people than the participating couple who did not provide informed consent, the following steps will be taken: First, participants will be advised to wear a small badge signaling that audio recording might happen as applied by Robbins et al [77,90]. Second, identity of anyone else than the partner will not be possible to be detected by the study personnel. Thus, anonymity will be granted.

Every study applying intensive longitudinal data assessment in people’s everyday life faces the challenge to potentially overburden participants resulting in high rates of refusal to participate or high attrition rates. There are studies using an intensive data assessment in diverse populations, demonstrating that studies similar to this one are feasible (eg, a study by Helgeson et al [91]); adolescent diabetes patients completed a 3- to 5-min questionnaire on the palm pilot every 2 hours throughout the day over a 2-day weekend, without increasing participants burden to the point of noncompliance). Another important challenge of this study regards the time-consuming and labor-intensive process involved in handling the large volumes of audio and video data. It is possible that as technology further develops, there will be the opportunity to make use of advanced speech recognition software; however, such software is not yet sophisticated enough to pick up on fineness of social interactions in romantic couple’s everyday life.

Using AAMC methodology will make a key contribution with regard to the objective operationalizations of social support, CDC, and physical activity, and thus, we will be able to provide detailed characterization of romantic couple’s communication about their dyadic diabetes management in daily life. To deepen the understanding of when social support and CDC are particularly effective, the data recorded with the AAMC will also be used to detect affect by a multimodal sensor fusion approach. The results of this study will provide a sound basis for the theory- and evidence-based development of dyadic interventions to change health behavior in the context of couple’s dyadic illness management. Implications may include exploring opportunities for the use of the AAMC methodology and inform other areas of couple’s everyday illness management of other chronic illnesses.

## Appendix

Multimedia Appendix 1 Existing peer-review reports from the Swiss National Science Foundation.

## Abbreviations

AAMC: ambulatory assessment application for the open-source behavioral intervention platform MobileCoach
CDC: common dyadic coping
EAR: electronically activated recorder
EMA: ecological momentary assessment
IS: information system
SEDC: system for coding dyadic coping
T2DM: type II diabetes mellitus
