# Supplementary material for: Social Support and Common Dyadic Coping in Couples' Dyadic Management of Type II Diabetes: Protocol for an Ambulatory Assessment Application
Source: JMIR Res Protoc. 2019 Oct 4;8(10):e13685. doi: 10.2196/13685 (PMC6802534; doi:10.2196/13685)
Supplement: Multimedia Appendix 1 [file resprot_v8i8e13685_app1.pdf]

www.snf.ch
Wildhainweg 3, Postfach 8232, CH-3001 Bern

Review 2

Application data

Project Title	Measuring the Impact of Social Support and Common Dyadic Coping on Couple’s Dyadic Management of Type II Diabetes by a Novel Ambulatory Assessment Application for the Open Source Behavioral Intervention Platform MobileCoach
Project title in English	Measuring the Impact of Social Support and Common Dyadic Coping on Couple’s Dyadic Management of Type II Diabetes by a Novel Ambulatory Assessment Application for the Open Source Behavioral Intervention Platform MobileCoach
Project number	CR12I1_166348
Instrument	Interdisciplinary project
Research Field	Social sciences
Main Discipline	10105 Psychology
Main Applicant	Urte Scholz
Amount requested (CHF)	666914

Comments regarding the overall assessment

The proposed project promises very important contributions from a practical point of view for individuals and health care systems dealing with one of the most serious chronic diseases of our days. The expected theoretical contributions are less apparent but, considering the scientific record and expertise of the research team, these could be important too.

Detailed evaluation

Applicants' scientific track record and expertise

Both the principal investigator and the two co-investigators have appropriate scientific track record and expertise for conducting the proposed project successfully. Their expertise is demonstrated by recently published or in press scientific work in top journals and conference proceedings.

Scientific relevance, originality and topicality

The project is relevant and original. Its practical usefulness for the possible support of diabetics with the help of mobile information technology tools is unquestionable. However, its theoretical background and, consequently, possible theoretical contributions are less apparent. Thus, some theoretical anchors (i.e. theories or models) regarding behavioral change and attitude or acceptance regarding information technology use should have been mentioned for better theoretical soundness. Open research questions mention as support for the necessity of this study a 1992 work and this may trigger some doubts. More recent references should have been provided.

Suitability of methods and feasibility

The methods described are appropriate, in general. The actual research questions look to broad and it may be difficult to fully answer them. Rather, for better focus, some factors of influence to be tested should have been identified, based on the theoretical background. The constructs to be actually measured and the approach of measuring them are not so clear for the reader. Recruiting participants from a variety of sources, as mentioned, may pose problems for sample homogeneity and participants' commitment to complete the study. Overall, the proposed study seems feasible but more details would have helped understanding.

Interdisciplinary aspect of the project

The project looks very well from the perspective of multi-disciplinary contribution. This is achieved by the manner the project was designed as well as by the background and expertise of the investigators. As mentioned above, a more solid theoretical support and more obvious expected theoretical contributions in at least one or two of the participating disciplines (e.g. healthcare, human behavior or information systems) would have improved the scientific quality.

Review 3

Application data

Project Title	Measuring the Impact of Social Support and Common Dyadic Coping on Couple’s Dyadic Management of Type II Diabetes by a Novel Ambulatory Assessment Application for the Open Source Behavioral Intervention Platform MobileCoach
Project title in English	Measuring the Impact of Social Support and Common Dyadic Coping on Couple’s Dyadic Management of Type II Diabetes by a Novel Ambulatory Assessment Application for the Open Source Behavioral Intervention Platform MobileCoach
Project number	CR12I1_166348
Instrument	Interdisciplinary project
Research Field	Social sciences
Main Discipline	10105 Psychology
Main Applicant	Urte Scholz
Amount requested (CHF)	666914

Comments regarding the overall assessment

Given my comments in the previous sections of this proposal, I rate this proposal as [redacted] for the following reasons. The team assembled is top notch, but would benefit from a member with expertise in T2DM. The proposed investigation is important and will certainly make major contributions to the understanding of support behavior in couples and novel assessment strategies using cutting edge technologies. Yet, the conceptual underpinning of the investigation would benefit from a compelling theoretical perspective to guide the researchers' thinking regarding the interrelation of visible and invisible support and common dyadic coping. The proposal is truly outstanding in regard to its interdisciplinarity and innovative methodological approach.

Detailed evaluation

Applicants' scientific track record and expertise

Urte Scholz: Dr. Scholz's CV clearly indicates prolific scholarly output in the past 5 years, with 48 peer-reviewed publications listed, most of which are in high impact journals. In terms of her publications, my only concern is that only 8 are first authored papers. But her publishing record certainly highlights her ability to successfully collaborate and mentor students. I also think Dr. Scholz is prominent in her field, as evidenced by her serving as president of the Health Psychology Division of the International Association of Applied Psychology, serving on a variety of editorial boards for top health psychology journals, and reviewing for a variety of journals.

Guy Bodenmann: Dr. Bodenmann is a worldwide authority on support within couple relationships. I can certainly think of no better co-applicant than Dr. Bodenmann for such a project. He has published 45 peer-reviewed articles in the past 5 years (7 as first author), not to mention a number of books and book chapters. His work continues pushing the field forward, in terms of how couple relations are intertwined in personal well-being, and his intervention approach (CCET) is increasingly being recognized as an efficacious treatment for couple distress and even depression.

Elgar Fleisch: Dr. Fleisch boasts extensive academic and industry experience, having founded and served on the board of numerous technology companies. In terms of scholarly contributions, Dr. Fleisch has 30+ publications in the previous 5 years, but none as first author. My field is not technology, so it is difficult for me to evaluate the quality of his scholarly output. But he, along with his team, are clearly contributing a great deal to our understanding of how technology can be leveraged to support behavior change.

Ability to carry out the project: This team has demonstrated a strong track record and understanding of couple support (Scholz & Bodenmann), couple management of chronic illness (Scholz & Bodenmann), and technology use for behavior change (Fleisch). The one thing missing from this team is someone with expertise of type 2 diabetes, in particular. Overall, a very strong team with more than enough capacity to complete the proposed project.

Scientific relevance, originality and topicality

Given the importance of better understanding ways in which contextual factors can be leveraged to improve diabetes outcomes coupled with the muddied understanding of whether support between intimate partners is helpful or not, this team has proposed a project that is extremely important to a number of academic disciplines. This focus would certainly be enough, but the additional aspect of pioneering innovative technological approaches for more accurate measurement is the proverbial "cherry on top." That being said, I also see some limitations in the proposal, which I will detail below.

The selection of the diabetes outcome variables was not well-justified and seems rather "thin." This is where the team would have benefited from having someone with expertise in T2DM. It seems that diabetes distress, efficacy, and perceived consequences of living with the illness are also relevant outcome variables, as is HbA1c as an objective measure of glycemic control.

Second, I certainly applaud investigation into how visible and invisible support and common dyadic coping are uniquely linked with diabetes outcomes, but the associations are likely much more complicated than that. These types of support are likely interrelated and the impact may be dependent on the presence or absence of other forms of support (moderation). This rather shallow level of conceptualization is likely due to the fact that there is no theoretical perspective being used to guide this study, as far as I could tell.

Finally, I agree that more research is needed to synthesize the disparate findings on support behaviors in couples, but this project does not propose to assess the support behaviors most commonly assessed in the chronic illness research. A scale developed by Buunk (1996) and extensively used by Hagedoorn is the most frequently occurring measure of social support (active engagement, protective buffering, and overprotection) in the couples and health literature (from my reading). As such, the project will make a bigger contribution to the broader literature on social support processes within couple relationships than couple support in the context of chronic illness.

Suitability of methods and feasibility

I think the methods chosen are excellent in their ability to answer the research questions. I do not have expertise in the technological aspects of this project and cannot comment on the feasibility of preparing the MobileCoach app for data collection. But the other aims of the project are certainly feasible within the scope of this project.

Interdisciplinary aspect of the project

This proposal is an exemplar for high-quality interdisciplinary work, seamlessly blending expertise from psychology, information systems research, and computer science.

www.snf.ch
Wildhainweg 3, Postfach 8232, CH-3001 Bern

Review 4

Application data

Project Title	Measuring the Impact of Social Support and Common Dyadic Coping on Couple’s Dyadic Management of Type II Diabetes by a Novel Ambulatory Assessment Application for the Open Source Behavioral Intervention Platform MobileCoach
Project title in English	Measuring the Impact of Social Support and Common Dyadic Coping on Couple’s Dyadic Management of Type II Diabetes by a Novel Ambulatory Assessment Application for the Open Source Behavioral Intervention Platform MobileCoach
Project number	CR12I1_166348
Instrument	Interdisciplinary project
Research Field	Social sciences
Main Discipline	10105 Psychology
Main Applicant	Urte Scholz
Amount requested (CHF)	666914

Comments regarding the overall assessment

An outstanding team of investigators is assembled to conduct the proposed investigation of social influence on disease management, a topic of crucial public health interest. The proposed investigation has several notable strengths including the multi-method study design, the focus on a patient population of couples managing Type 2 diabetes and the assessment of both partners and their interaction patterns in their natural setting. The proposed study is somewhat limited by the emphasis on independent, rather than interactive, effects of the key social indicators of visible and invisible support and dyadic coping. Additionally, the proposed investigation could be strengthened by considering the potential for unique associations of the key social interactions with different health behaviors and by more fully elaborating the relevance and measurement of selected health outcomes in the context of diabetes and its management.

Detailed evaluation

Applicants' scientific track record and expertise

Each of the applicants has a strong record of peer-reviewed publications and scientific communication reflecting a programmatic line of research of direct relevance to the current application.
Dr. Scholz currently holds the position of Professor of Applied Social and Health Psychology at the University of Zurich. Her work consistently has included a key social partner in the investigation of health promotion, disease management, and behavior change among individuals across the lifespan. The role of social context in health is of vital importance in understanding individual’s efforts to engage in healthy lifestyle behaviors and to adhere to medical recommendations. Her publications over the past five years address partners’ behaviors such as providing support (with and without the awareness of the intended recipient), and exerting social control across a variety of health contexts. Specifically, partners’ support and control have been examined in the health behavior change process of several behaviors including smoking cessation, physical exercise, consuming a low-fat diet, and cancer screening. Her innovative work has been published in several leading health journals such as Health Psychology, Social Science and Medicine, BMC Public Health, and in both US and European outlets. Dr. Scholz also has been activity involved in scientific communication through membership and leadership in several organizations such as the International Association of Applied Psychology. She also has provided expert review of journal manuscripts and grant proposals for organizations in Europe and the US.
Dr. Bodenmann currently holds the position of Professor of Clinical Psychology for Children/Adolescents and Couples/Families at University of Zurich. He is internationally recognized for his contributions to understanding dyadic coping among couples in the context of stress. Over the past five years, he has produced a large body of work that addresses partners’ communication during conflict and stressful events, particularly their dyadic coping and its association with their individual health and well-being and the quality of their relationship. This work is published in a wide variety outlets in the US and Europe and in top-tier journals such as Journal of Consulting and Clinical Psychology, Psychological Science, Journal of Family Psychology, Swiss Journal of Psychology, and British Journal of

Health Psychology. Dr. Bodenmann is a member of several scientific organizations in the US and in Europe including German Association of Psychologists, Swiss Association of Psychologists, American Psychological Association, and the National Council on Family Relations.
Dr. Fleisch currently holds the position of Professor of Information and Technology Management. Among his strong record of publications and scientific communication are papers focused on the use of technology in health behavior interventions of direct relevance to the current proposal. One such paper entitled “MobileCoach: A Novel Open Source Platform For The Design Of Evidence-Based, Scalable And Low-Cost Behavioral Health Interventions – Overview and Preliminary Evaluation in the Public Health Context was recently recognized with an Outstanding Paper Award and Best Graduate Student Paper Award at a Telecommunications Symposium in New York.

This outstanding team of investigators clearly has the expertise necessary to successfully carry out the proposed project. Drs. Scholz and Bodenmann have considerable expertise in the areas of social support and dyadic coping that are central to this investigation. Importantly, each has successfully completed multiple large-scale investigations of the associations of support and dyadic coping with health and well-being similar to the project currently proposed. Dr. Fleisch brings expertise in the development and utilization of mobile technology to aid behavioral health interventions under investigation in the proposed study. Although this team does not have a record of prior collaboration, each investigator has substantial experience working as part of a research team as can be seen in their records of collaborative research publications.

Scientific relevance, originality and topicality

The proposed project has high scientific relevance for public health research and practice. A considerable strength of the study design is the focus on the role of a key social partner in the management of Type 2 diabetes. It increasingly is recognized that patients with chronic disease do not manage their disease in isolation, but rather rely on close social partners for support and aid to properly carry out the complex daily regimen that often accompanies a chronic disease diagnosis. More specifically, the proposed study has potential to advance understanding of the role of social partners in promoting (and possibly, in hindering) disease management through partners’ social interaction patterns and behaviors through the investigation of both visible and invisible support and dyadic coping. This study will provide needed information regarding social interaction patterns that facilitate treatment adherence and circumstances under which some types of social interactions may be more (and less) beneficial for behavioral adherence. Of particular note, the proposed project has potential to develop a technological tool to aid the collection and analysis of data from social interactions that can inform research and practice on strategies to promote health behavior change and maintenance.

The proposed project is original in several aspects of study design. The investigation of social support together with dyadic coping in a patient population, specifically the management of diabetes, is a unique feature of the current proposal. Moreover, an understudied form of support, invisible support, is addressed in the proposed project. As previously noted, the project also utilizes an innovative data collection tool to capture multiple behaviors (physical activity, interpersonal interactions) and thereby reduce burden on participants who otherwise would be asked to manage several data capture devices during the study period. In addition, the project proposed will include multiple methods to capture the focal dyadic interactions through experience sampling, observation, and end-of-day diaries.

The central focus of the proposed investigation, social influence on disease management, is of strong public health interest. Adopting and maintaining healthy lifestyle behaviors has been shown to be challenging, even when necessary for the proper management of chronic disease such as diabetes. Identifying factors that promote behavioral adherence will reduce the personal and financial costs of chronic disease management for patients and their families. Given the recognized involvement of partners in the health care and disease management of patients, there is considerable need for investigations that advance understanding of conditions under which their involvement is beneficial (and when it can be intrusive and detrimental) to the behavioral adherence of patients managing chronic illness.

Suitability of methods and feasibility

The multi-method approach developed for examining social support and dyadic coping among couples managing Type 2 diabetes is a significant strength of this proposal. Data will be captured through experience sampling techniques, observation and coding, and daily diaries. The assessment of both partners and their interaction patterns in their natural setting is another strength of the proposed study design and methods. It merits mention, however, that the methods and tools designed for the experience sampling of partners’ support and dyadic coping allow for assessment primarily of verbal expressions that can be captured by the proposed audio recordings. Other expressions of support

and dyadic coping such as nonverbal communication that may accompany and underscore the verbal expressions will not be accounted for with the audio recordings. In particular, “invisible support” is likely to be conveyed without explicit verbal communication as noted on page 3 of the proposal “one partner takes care of unexpected housework –without telling – the other.” Thus, the utility of the audio recording for assessing invisible support between partners requires additional clarification. Although nonverbal communication of support and dyadic coping may be assessed through the video recorded observations and daily diaries, these types of expressions are not given sufficient attention in the current proposal.

The proposed aims and hypotheses also are somewhat limited in that the focus of the study is on main effects of visible and invisible support and dyadic coping. Broadening the aims to include the association of each type of support and dyadic coping in the context of the others (potential moderation) is likely to result in greater understanding of the association of partners’ interactions and disease management. Additionally, the interactions of partners assessed as visible and invisible support and dyadic coping, are hypothesized to operate in the same way for all three health behaviors; physical activity, diet adherence, and medication adherence. That is, visible support and dyadic coping are “assumed to be positively associated with patients’ diabetes-related health behaviors” and “invisible support is assumed to be negatively related to patients’ diabetes-related health behaviors.” It merits consideration whether the associations of partners’ interactions may depend on the target health behavior. For instance, invisible support may benefit diet adherence through activities such as shopping for and preparing healthy foods. It also should be noted that the expectation that invisible support will be negatively related to health behaviors is based on limited empirical evidence. Further, the recruitment of both male and female patients with diabetes and their partners should be addressed in order to adequately address the hypotheses that gender moderates the association between visible support and health outcomes.

Overall, partners’ interaction patterns and the assessment of these interactions seem more central to the study design and analyses than the associations of these interaction patterns with proper disease management behaviors, i.e., physical activity, diet and medication adherence. The rationale and methods for assessing visible and invisible support and dyadic coping are more innovative and more carefully elaborated than is the rationale and measurement of behavioral adherence outcomes. The methods designed for the proposed investigation have strong potential to advance knowledge of partners’ interactions, particularly visible and invisible support and dyadic coping, in the context of diabetes management. The potential for advancing understanding of linkages among these interaction patterns and health outcomes among patients with diabetes would be strengthened by refining the study hypotheses for each type of health behavior and by using more objective measures of the health behaviors in the proposed investigation.

The feasibility of the proposed project is addressed carefully in this application. Given the expertise of the investigators and collaborators and their collective success with similar research projects, it is highly likely that the goals of the application can be reached in the time proposed and with the resources requested. A potential concern with the feasibility of the proposed investigation, however, is the likelihood of capturing partners’ exchanges about disease management through the experience sampling method. It is not clear how frequently partners openly discuss aspects of behavioral adherence and whether the proposed audio recording assessment procedure in the one week time frame will adequately represent these interactions. As previously noted, though, the multi-method study design will provide alternative assessments of visible and invisible support and dyadic coping through daily diaries and video-recorded observations.

Interdisciplinary aspect of the project

Investigation of social influence on health promotion and disease management is strengthened by interdisciplinary collaboration to address aspects of interpersonal relationships, health behavior change and maintenance, and disease processes. The proposed team of investigators includes social scientists with demonstrated expertise in interpersonal relationship dynamics and experience in investigating health behavior change. The novel multi-method study design is facilitated by including expertise from Information and Technology Management for capturing physical activity and social interactions of participants anticipated to be associated with behavioral adherence and psychological well-being in the context of chronic illness. The interdisciplinarity of the project could be extended to include a health care professional with expertise in the role of lifestyle behaviors and medication adherence in the management of diabetes to contribute to assessment and interpretation of key indicators of diabetes management.

www.snf.ch
Wildhainweg 3, Postfach 8232, CH-3001 Bern

Review 1

Application data

Project Title	Measuring the Impact of Social Support and Common Dyadic Coping on Couple's Dyadic Management of Type II Diabetes by a Novel Ambulatory Assessment Application for the Open Source Behavioral Intervention Platform MobileCoach
Project title in English	Measuring the Impact of Social Support and Common Dyadic Coping on Couple's Dyadic Management of Type II Diabetes by a Novel Ambulatory Assessment Application for the Open Source Behavioral Intervention Platform MobileCoach
Project number	CR12I1_166348
Instrument	Interdisciplinary project
Research Field	Social sciences
Main Discipline	10105 Psychology
Main Applicant	Urte Scholz
Amount requested (CHF)	666914

Comments regarding the overall assessment

Strengths: researchers' background and expertise; degree of interdisciplinarity involved in the project; topic in which more research is needed; novel approach to assess social interactions in situ; development of an open source mobile technology

Weaknesses: researchers' lack of expertise in psychosocial factors related to diabetes management and research with older adults; frequency with which participants are being audio-recorded (compared to frequency of occurrence of social interactions examined); other measurement concerns (e.g., social support, medication adherence); potential sample bias

Detailed evaluation

Applicants' scientific track record and expertise

All three applicants have impressive track records in the past 5 years in their respective areas of expertise. They are all extremely prolific and publish in well-regarded journals. Dr. Scholz, in particular, has an impressive history of research funding. The degree of science communication and networking is also good for all three applicants. Although there is no history of previous collaborations with one another, they all contribute a unique area of expertise to the proposed project (Dr. Scholz in social support, health behaviors, and experience sampling methodology; Dr. Bodenmann in dyadic coping among couples; and Dr. Fleisch in technology-supported behavior change). The team appears to have the expertise needed to carry out the project successfully. Expertise in psychosocial factors important for diabetes management or in older adults (who comprise most of individuals with type 2 diabetes) is missing, however.

Scientific relevance, originality and topicality

Given the central role that spouses play in their partners' diabetes management, this proposal is relevant to the field of health psychology (and related areas) and has the potential to increase knowledge and understanding of how couples manage the health behavior component of a regimen-intensive chronic condition that is becoming increasingly more common worldwide. The methods proposed are innovative and address a gap in the current literature on this topic.

Specifically, the proposed use of a mobile technology to assess couples' actual conversations and corresponding emotional responses (including valence and arousal) in their natural environment is unique and contributes to a better understanding of how couples interact with each other in managing diabetes. The proposal also seeks to better understand the construct of invisible support, which until recently, has only been assessed in an indirect way. There

are only a few concerns about the proposal's scientific relevance.

Some of the research cited in the background reflects another important type of social interaction that spouses commonly engage in to help their partners manage the health behavior component of diabetes (social control), yet the researchers refer to it as social support (Dr. Scholz actually has published on this topic). Social control is an understudied, yet important component, of couples' diabetes management.

The researchers do not provide background information for their hypothesis related to affective arousal (hypothesis 3.2). In fact, there is very little discussion about the literature on affective responses to support, in general (despite the relatively large body of literature on this topic).

Suitability of methods and feasibility

The methods proposed are appropriate for the research questions of interest, and the scope of the project is appropriate, given the timeline, personnel, and funds. I have some comments and concerns about the methods, however.

Although the attempt to measure supportive interactions in a more objective way is certainly admirable, my biggest concern with the proposed methodology is that the researchers are only sampling 5 minutes per hour for a few hours daily for 7 days (before 8am in the morning and four hours in the evening – and all weekend). Given the focus of the proposal on examining three different types of marital interactions, how likely is it that the researchers will capture each of these types of interactions with enough frequency – if at all?

The researchers note the importance of using objective measures of social interactions and physical activity, yet they are relying on self-report measures for medication adherence (for which there are currently objective measures available).

For the measure of social support, the researchers note that they will ask participants the following questions: "Today, I received emotional/instrumental support from my partner" and "Today, I provided emotional/instrumental support to my partner." How likely is it that laypeople will understand the difference in the types of support included in these questions?

Very little information about the psychometric properties of the scales being used is included.

If most of the participants are recruited from one clinic (but some are recruited from the community), there may be concerns about bias in the sample as a result of provider (or clinic) characteristics (vs. if the sample was only from one clinic or only community-based).

Interdisciplinary aspect of the project

The research questions in the proposed project require an interdisciplinary approach and the researchers have expertise in each of the areas required to successfully complete the project (Dr. Scholz has expertise in health psychology, Dr. Bodenmann has expertise in clinical psychology and Dr. Fleisch has expertise in information technology as it relates to health). Each of these disciplines is appropriately reflected in the organization of the project.

www.snf.ch
Wildhainweg 3, Postfach 8232, CH-3001 Bern

Review 5

Application data

Project Title	Measuring the Impact of Social Support and Common Dyadic Coping on Couple's Dyadic Management of Type II Diabetes by a Novel Ambulatory Assessment Application for the Open Source Behavioral Intervention Platform MobileCoach
Project title in English	Measuring the Impact of Social Support and Common Dyadic Coping on Couple's Dyadic Management of Type II Diabetes by a Novel Ambulatory Assessment Application for the Open Source Behavioral Intervention Platform MobileCoach
Project number	CR12I1_166348
Instrument	Interdisciplinary project
Research Field	Social sciences
Main Discipline	10105 Psychology
Main Applicant	Urte Scholz
Amount requested (CHF)	666914

Comments regarding the overall assessment

The proposed research has the potential to make novel and important theoretical, empirical, methodological, and technological contributions to our understanding of the social context of chronic illness management. It does so with reference to an illness that is burgeoning world-wide, type 2 diabetes. For these reasons, the scientific importance and practical implications of the proposed research are very considerable.

Detailed evaluation

Applicants' scientific track record and expertise

The applicants have demonstrated expertise and a proven track record that is relevant to the proposed research. This expertise includes extensive experience investigating health-behavior change, social support processes, and dyadic coping processes. Moreover, the applicants have expertise in recruiting couples and in conducting intensive assessments of individuals and dyads, using experience sampling methods, longitudinal methods, and experimental designs. Together with their collaborators, the applicants have impressive experience, as well, with the development and use of emerging technologies for ambulatory assessment of naturally occurring speech, physical activity and other health behaviors. They also have experience with the statistical analysis of complex data structures, using multilevel modeling and other methods for the analysis of data (including dyadic data) derived from intensive, short-term longitudinal studies. The applicants are very productive scholars with an impressive record of publications and previous successful grants. Considered together, the research team has the collective expertise and qualifications to undertake the proposed research.

Scientific relevance, originality and topicality

The proposed study of social support and common dyadic coping in the context of type 2 diabetes will have substantial scientific relevance to the field of health psychology, and to specializations within the fields of medicine and nursing that are concerned with the management of diabetes and, more broadly, the management of chronic illness. The management of many chronic illnesses occurs in the context of close relationships that affect, and are affected by, the challenges, successes, and setbacks encountered during the course of a family member's efforts to manage his or her chronic illness. By distinguishing between visible and invisible social support, and by evaluating the dyadic nature of couples' efforts to cope with a stressor, such as a chronic illness, the proposed research will bring greater coherence to our understanding of the social context of health-behavior change. The proposed research has great potential to reconcile conflicting findings in the literature regarding the effects of social support, and to add to our understanding of the types and circumstances of support that are most likely to benefit recipients. The proposed research will extend knowledge of the emerging, but still understudied, distinction between visible and

invisible social support by identifying particular kinds of transactions in naturally occurring and laboratory-based social interactions that reflect visible versus invisible support. Such knowledge, in turn, will help to inform our understanding of why visible versus invisible social support may exhibit entirely different associations with outcomes of interest, such as patients' treatment adherence and emotional well-being.

An exciting and innovative feature of the proposed research will be the analysis of naturally occurring speech samples derived over a 7-day period from the use of electronically activated recorder (EAR) technology to investigate not only the content, but also the affective quality, of such speech samples. Visible support, for example, might have manifestly supportive verbal content but a subtly critical affective tone and, if so, this would help to explain why visible support is sometimes associated with lower well-being in recipients. Invisible support, in contrast, may entail supportive content that does not detract from recipients' well-being either because recipients do not detect the communication as an explicit message of support or because the communication is rendered in way that is affectively neutral or positive, avoiding detrimental effects on recipients' well-being. Documenting such subtly different, but important, dimensions of couple's support-related communications will be extremely valuable in clarifying the particular kinds of transactions that underlie visible versus invisible support, and it will help to inform interventions to strengthen and improve couples' support-related communications. Such knowledge is particularly important in the context of chronic illness because support-related communications unfold regularly over many years, and spouses who become impatient over time with an ill partner's inconsistent or poor treatment adherence may not realize that their communications have become subtly critical or demanding. Because this study will be among the first to examine visible versus invisible support in the context of chronic illness, in concert with common dyadic coping, it will add valuable information and novel theoretical connections to a sparse knowledge base.

Another novel contribution of the proposed research is the integration of data derived an observational, laboratory-based study of support- and coping-related communications with data derived from the ambulatory assessments of such communications as they naturally occur in participants' lives. Collectively, these methods will allow for both objective and subjective assessments of key support- and coping-related processes in ways that will overcome limitations of self-report methods and will produce exceptionally rich data for examining connections between these processes and health behaviors that are crucial to managing type 2 diabetes.

Methodological contributions of the study are also important and are likely to inspire further research. The investigators' elaboration and novel applications of the EAR and Mobile Coach technologies for the ambulatory assessment of naturally occurring support, dyadic coping, and health behavior (including objectively assessed physical activity) are likely to be of great interest to researchers and to prompt future applications of these technologies to investigate relevant interpersonal processes in other illness contexts. The integration of the ambulatory data with data on parallel theoretical constructs from the observational study is also a novel feature of the study that is likely to inspire a similar approach in future studies.

The applicants have state-of-the-art knowledge of the relevant theoretical literatures that will guide the proposed research and the cutting-edge methods that will be used to collect and analyze their data.

The applicants are bringing their very substantial expertise to bear on a topic of considerable societal importance, as rates of type 2 diabetes are burgeoning worldwide, and the management of type 2 diabetes often occurs in a social context (with effects that can be either helpful or unhelpful). As noted above, the proposed research has important broader impacts in that it can inform health-care practitioners and individuals and their family members who are coping with chronic illness about the kinds of illness-related communications that are most versus least likely to be helpful in fostering successful illness management. The applicants are clearly skilled in disseminating their research, which increases confidence in the anticipated broader impacts of the research.

Suitability of methods and feasibility

The methods are well-suited to address the study aims. The methods are innovative and will capture both naturally occurring and laboratory-based communications that reflect health-related support and coping. One potentially minor limitation of the ambulatory assessments (using the EAR technology) is that may be particularly well-suited to capturing verbal forms of support and coping, but less well-suited to capturing nonverbal forms of visible and invisible support. Spouses' efforts to convey support though a sympathetic facial expression or touch that are unaccompanied by a verbal comment may not be detected by the EAR methodology. Similarly, spouses' nonverbal actions that are meant to support their ill partner's treatment adherence, such as removing unhealthy foods from other house or laying out medications to be taken outside of the ill partner's awareness, are also unlikely to be detected by the EAR methodology. The data from the observational study that are meant to complement the ambulatory data derived from the EAR methodology should be able to detect nonverbal, as well as verbal, forms of support that occur during a structured dyadic interaction; like the ambulatory methods, however, the observational

methods may be less able to detect nonverbal forms of support that occur outside the context of verbal exchanges between members of the couple. Some, though not all, forms of invisible support may be likely to fall into the latter category (as speculated, but not yet examined in the literature). This is a minor reservation that has a bearing only on the forms of invisible support to which the applicants' results may be likely to generalize. This reservation is minor in view of the exceptionally rich data that the proposed research will generate on social support processes, dyadic coping, and treatment adherence and in view of the strongly likelihood that most support processes in couples are verbal in nature.

Another minor concern is that spouses who are seeking to make supportive comments (as assessed either via the ambulatory methods or the laboratory discussion task) may make "we" statements that reflect a collaborative (dyadic) approach to the ill partner's illness management. Such we statements would seem to overlap with common dyadic coping to some extent. Therefore, a plan will be needed regarding how to treat such potentially overlapping statements in analyses that are meant to examine the relative contributions of support and common dyadic coping to patients' illness management.
An additional minor concern is that the observational study, by requiring participants to engage in a structured conversation about concerns related to the ill individual's diabetes management, seems somewhat less likely to produce instances of invisible support. Comments made by the non-ill spouse that are meant to be supportive but are completely outside of the ill partner's awareness (one meaning of invisible support) seem likely to be rare. Comments made by the non-ill spouse that are meant to be supportive but are not encoded as supportive to the same extent by the ill partner could reflect another meaning of invisible support, but they could also reflect the ill partner's dissatisfaction with the support provided. Alternatively, such comments could be experienced (unconsciously) by the ill partner as supportive/beneficial but might not by encoded as support per se. An example, drawing from the work of Bolger and Amarel (2007), would be a statement intended to preserve the ill-partner's sense of self-efficacy in which the spouse imagines aloud that (s)he would have great difficulty making a particular health behavior change but recognizes its importance. Such a statement might help an ill partner who is struggling with adherence feel less bad about his or her difficulties and might motivate continued effort, but it would not necessarily be encoded as support (and hence would reflect invisible support). I believe that the applicants will be able to tease apart these different meanings and ways of operationalizing invisible (and visible) support, but it will require careful work to do so.

The eligibility criteria for inclusion in the study do not include the duration of the diagnosis of type 2 diabetes. Recently diagnosed patients are likely to have different kinds of interactions with their partners than are patients who have had type 2 diabetes for many years. Support processes and patterns of dyadic copying may change over time and, although the applicants do plan to treat duration of the illness as a covariate, they might want to consider treating it as an eligibility criterion, particularly since the sample size may limit subgroup analyses based on duration (should illness duration vary extensively across participants and prove to be related to key variables).

The project timeline and milestones seem realistic. Overall, I would judge the project to be feasible given the resources requested.

Interdisciplinary aspect of the project

The proposed research project does require an interdisciplinary approach, and the research team reflects the relevant disciplines for the project (psychology, information systems research, quantitative methods). The contributions of team members from different disciplines are carefully integrated in the proposal, and the advancement of young academics seems appropriate, as well.
